# Symptoms of depression are associated with reduced leisure-time physical activity in adult individuals with type 1 diabetes

**DOI:** 10.1007/s00592-021-01718-6

**Published:** 2021-05-19

**Authors:** Aila J. Ahola, Heidi Tikkanen-Dolenc, Carol Forsblom, Valma Harjutsalo, Per-Henrik Groop, on behalf of the FinnDiane Study Group

**Affiliations:** 1 Folkhälsan Research Center, Folkhälsan Institute of Genetics, Helsinki, Finland; 2 Department of Nephrology, University of Helsinki and Helsinki University Central Hospital, Helsinki, Finland; 3 Research Program for Clinical and Molecular Metabolism, Faculty of Medicine, University of Helsinki, Helsinki, Finland; 4 National Institute for Health and Welfare, Helsinki, Finland; 5 Department of Diabetes, Central Clinical School, Monash University, Melbourne, VIC Australia

**Keywords:** Beck Depression Inventory, Leisure-time physical activity, Symptoms of depression, Type 1 diabetes

## Abstract

**Aims:**

Here, we investigated the association between depressive symptoms and leisure-time physical activity (LTPA) in type 1 diabetes.

**Methods:**

Data from adult individuals with type 1 diabetes without evidence of diabetic kidney disease or macrovascular complications, participating in the Finnish Diabetic Nephropathy Study, were included. Based on a questionnaire, weekly LTPA as metabolic equivalent of task hour was calculated. Activity levels (inactive, moderately active, active), weekly frequencies (< 1, 1–2, > 2), intensities (low, moderate, high), and single session durations (< 30, 31–60, > 60 min) were assessed. Depressive symptomatology was evaluated using the Beck Depression Inventory (BDI). We calculated a continuous BDI score and divided participants into those with (BDI score ≥ 16) and without (BDI score < 16) symptoms of depression. For sensitivity analyses, we additionally defined symptoms of depression with antidepressant agent purchases within a year from the study visit.

**Results:**

Of the 1339 participants (41.7% men, median age 41 years), 150 (11.2%) reported symptoms of depression. After adjustments, both higher BDI scores and depressive symptomatology were associated with more inactive lifestyle, and lower frequency and intensity of the LTPA. The BDI score was additionally associated with shorter single session duration. For antidepressant purchases, lower odds were observed in those with higher intensity and longer single session duration of LTPA.

**Conclusions:**

Depressive mood is harmfully related to LTPA in type 1 diabetes. In order to improve the long-term health of individuals with type 1 diabetes, efforts to increase both mental well-being and physical activity should be taken.

**Supplementary Information:**

The online version contains supplementary material available at 10.1007/s00592-021-01718-6.

## Introduction

Regular physical activity is known to bring about considerable health benefits [1]. Therefore, it has been recommended that most adults with diabetes weekly engaged in moderate-to-vigorous intensity activity for a duration of at least 150 min, spread over at least 3 days with no more than 2 consecutive days without activity [2]. In individuals with type 1 diabetes, physical activity has shown, not only to enhance physical fitness, but also to improve glycaemic control, body composition, lipid levels, endothelial function, and insulin sensitivity [3, 4]. There is also increasing evidence that physical activity reduces the risk of long-term vascular complications, commonly associated with type 1 diabetes [5–7], and that of premature mortality [8, 9].

Despite the known benefits, the levels of physical activity are frequently suboptimal, and as many as > 60% of individuals with type 1 diabetes have been observed to fall below the physical activity recommendations [10]. Among the important predictors of physical inactivity, in the general population, is depression [11]. However, the association between depressive mood and physical activity has not been extensively investigated in individuals with type 1 diabetes. In one of the identified studies, depressive symptomatology was associated with increased odds of reporting no physical activity and decreased odds of reporting ≥ 5 weekly physical activity sessions [12]. In another study of adult participants in the T1D Exchange clinic registry, individuals with symptoms of depression were observed to exercise less often than those without [13]. Common to both of the above studies, beyond frequency, no data were available on other aspects of physical activity. Importantly, we have previously shown that, for example, intensity and duration of physical activity play important roles in the health of individuals with type 1 diabetes [5, 9, 14].

Our aim was therefore to investigate the association between symptoms of depression and leisure-time physical activity (LTPA), measured as total amount of physical activity, activity level, and frequency, intensity, and single session duration, in a large sample of adult individuals with type 1 diabetes. According to our hypothesis, symptoms of depression are associated with lower overall activity level.

## Methods

Study subjects were individuals with type 1 diabetes taking part in the Finnish Diabetic Nephropathy (FinnDiane) Study. The FinnDiane Study is an ongoing endeavour to reveal risk factors for long-term diabetes complications in type 1 diabetes. Since its launch in 1997, more than 5000 individuals have been nationally recruited at 92 sites. Type 1 diabetes was defined as diabetes onset before the age of 40 years and permanent insulin treatment initiated within a year from the diagnosis. In the current analyses, we included all FinnDiane Study participants with completed Beck Depression Inventory (BDI) and LTPA questionnaires and estimated glomerular filtration rate (eGFR) ≥ 60 ml/min/1.73 m^2^. Individuals on dialysis or with a kidney transplant and those with a history of coronary events (acute myocardial infarction, coronary vascular procedure, stroke, amputation or peripheral vascular procedure) were excluded. Data were collected between January 2004 and February 2020. The Ethics Committee of Helsinki and Uusimaa Hospital District approved the study protocol. The study was carried out in accordance with the Declaration of Helsinki and its later amendments. Written informed consent was obtained from all individuals prior to study participation.

The procedures conducted during the FinnDiane Study visit have previously been reported [15]. Briefly, height and body weight were measured, and body mass index (BMI) was calculated. Following a minimum of 10-min rest, seated blood pressure was measured twice, and the mean of the measurements was calculated. Blood was drawn for central analyses of serum lipids, lipoproteins, and creatinine concentrations. Glycated haemoglobin (HbA_1c_) was measured locally using a standardized assay. The CKD-EPI equation by Levey et al. [16] was used to calculate eGFR. Current smoking was self-reported on a questionnaire. Data on dialysis, kidney transplant, and coronary events were obtained from standardized questionnaires and medical records.

Beck Depression Inventory (BDI) was used to estimate the symptoms of depression [17]. This self-report questionnaire includes 21 symptom-attitude categories including mood, pessimism, sense of failure, lack of satisfaction, guilty feeling, sense of punishment, self-hate, self-accusations, self-punitive wishes, crying spells, irritability, social withdrawal, indecisiveness, body image, work inhibition, sleep disturbance, fatigability, loss of appetite, weight loss, somatic preoccupation, and loss of libido. Each of these categories contains four to six statements of increasing symptom severity, of which participants chose the one that best describes their current situation. Upon completing the questionnaire, a total score ranging between 0 and 63 was calculated. Higher scores are indicative of more severe symptoms of depression. The BDI score was used as a continuous variable, and additionally classification into those with (BDI score ≥ 16) and without (BDI score < 16) symptoms of depression was made [18]. For sensitivity analyses, we additionally assessed symptoms of depression as purchases of antidepressant agents. These data were obtained from the Drug Prescription Register of the Social Insurance Institute of Finland. All individuals with purchases of drugs belonging to the classes N06A and N06CA (The Anatomical Therapeutic Chemical Classification System with Defined Daily Doses), within a year from the study visit, were considered having symptoms of depression.

LTPA was assessed using a validated self-report questionnaire, which was based on the form used in the Kuopio Ischemic Heart Disease Risk Factor study, an adaptation of the Minnesota LTPA questionnaire, as previously described [19]. In short, mean frequency, single session duration, and intensity of LTPA were reported for the previous 12 months for the 21 most common forms of physical activities in Finland. Based on these reports, total LTPA was calculated as weekly metabolic equivalent of task hours (METh). In addition to using the METh as continuous variable, we also categorized the participants into three groups of activity level (inactive, < 10 METh/week; moderately active, 10–40 METh/week; active, > 40 METh/week). Moreover, the following groupings were made: based on the reported mean frequency, < 1 weekly sessions, 1–2 weekly sessions, and > 2 weekly sessions; based on single session duration, ≤ 30 min, 31–60 min, and > 60 min; and based on the reported intensity of the LTPA, low-, moderate-, and high-intensity LTPA.

Categorical variables are presented as frequencies, and continuous variables with non-normal distribution are presented as medians (interquartile ranges). Between-group comparisons were conducted using Chi-squared test and Mann–Whitney U-test, respectively. To study the associations between depression variables and LTPA as continuous variable (METh), we used generalized linear regression analysis. Multinomial logistic regression analysis was used to study the associations between depression variables and categorical LTPA variables. In these latter analyses, those inactive (for activity), those reporting < 1 weekly LTPA session (for frequency), those reporting low-intensity LTPA (for intensity), and those reporting < 30 min of LTPA (for duration) were treated as reference groups. Models were adjusted for sex, age, BMI, eGFR, and current smoking. All analyses were conducted with IBM SPSS Statistics for Windows, version 25.0 (IBM Corp, Armonk, NY, USA). A two-tailed P-value < 0.05 was chosen to designate statistical significance.

## Results

Data were available from 1339 participants (41.7% men, median [interquartile range] age 41 [33–51] years). In all, 150 (11.2%) participants reported BDI scores ≥ 16. These individuals with symptoms of depression were more frequently women and had higher total cholesterol concentration compared to those with no symptoms of depression (Table 1).

**Table 1:** Basic characteristics of the study sample divided by depression status

	BDI score < 16 *n* = 1189 (88.8%)	BDI score ≥ 16 *n* = 150 (11.2%)	*P*
BDI score	3 (1 – 7)	22 (18 – 27)	< 0.001
Men, %	43.2	29.3	0.001
Age, years	41 (33 – 51)	41 (32 – 52)	0.877
Smoker, %	16.2	22.3	0.079
Body mass index, kg/m^2^	25.3 (23.1 – 27.9)	25.6 (23.3 – 29.4)	0.160
SBP, mmHg	134 (123 – 145)	133 (121 – 147)	0.552
DBP, mmHg	78 (72 – 85)	80 (74 – 85)	0.117
HbA_1c_, mmol/mol	64 (55 – 72)	64 (57 – 76)	0.177
HbA_1c_, %	8.0 (7.2 – 8.7)	8.0 (7.4 – 9.1)	0.177
Total cholesterol, mmol/l	4.6 (4.1 – 5.2)	4.8 (4.3 – 5.4)	0.039
HDL cholesterol, mmol/l	1.56 (1.31 – 1.88)	1.56 (1.31 – 1.90)	0.687
Triglycerides, mmol/l	0.9 (0.7 – 1.3)	0.9 (0.7 – 1.4)	0.593

Data are presented as frequencies for categorical variables, and medians (interquartile ranges) for continuous variables with skewed distribution. Between-group comparisons were done with Chi-squared test, and Mann–Whitney U-test, respectively. BDI, Beck Depression Inventory; BMI, body mass index; SBP, systolic blood pressure; DBP, diastolic blood pressure

Individuals with depressive symptomatology reported lower levels of total LTPA compared to those without (13.2 vs. 19.8 METh, *P* < 0.001, Table 2). In addition, the two groups significantly differed with respect to the reported activity levels, frequencies, and intensities of LTPA. Here, those with symptoms of depression more frequently reported being inactive (42.2% vs. 23.7%), taking part in LTPA less often than once a week (30.8% vs. 17.7%), and taking part in low-intensity LTPA (43.1% vs. 21.4%). No difference in the reported single session duration was evident between those with and without symptoms of depression.

**Table 2:** Leisure-time physical activity divided by depression status

	BDI score < 16 *n* = 1189 (88.8%)	BDI score ≥ 16 *n* = 150 (11.2%)	*P*
Total LTPA, METh/week	19.8 (10.6 – 34.1)	13.2 (6.6 – 28.4)	0.001
Activity level			< 0.001
Inactive	23.7	42.2	
Moderately active	58.4	42.2	
Active	17.9	15.6	
Frequency of LTPA			< 0.001
< 1 session per week	17.7	30.8	
1–2 sessions per week	22.8	25.9	
> 2 sessions per week	59.5	43.3	
Intensity of LTPA			< 0.001
Low intensity	21.4	43.1	
Moderate intensity	54.3	45.8	
High intensity	24.3	11.1	
Duration of LTPA session			0.063
< 30 min	11.0	17.5	
31–60 min	56.4	55.5	
> 60 min	32.6	27.0	

Data are presented as medians (interquartile ranges) or frequencies. Between-group comparisons are conducted with Mann–Whitney U-test and Chi-squared test, respectively. BDI, Beck Depression Inventory; LTPA, leisure-time physical activity; METh/week, metabolic equivalent of task hours per week; inactive, reported weekly LTPA < 10 METh; moderately active, reported weekly LTPA 10–40 METh; Active, reported weekly LTPA > 40 METh; low intensity, no shortness of breath and no sweating; moderate intensity, moderate degree of shortness of breath and sweating; high intensity, severe shortness of breath and profuse sweating

After adjusting for age, sex, BMI, eGFR, and current smoking, total physical activity as METh was not associated with the BDI score (Table 3). Instead, being moderately active (0.966 [0.950–0.983], *P* < 0.001) or active (0.975 [0.953–0.998], *P* = 0.037), as compared to being inactive, was associated with lower BDI score after the same adjustments. Relative to reporting LTPA less frequently than once a week, those with more than two weekly sessions had lower BDI score (0.966 [0.947–0.984], P < 0.001). Additionally, individuals reporting above low-intensity LTPA had lower BDI scores (moderate intensity, 0.960 [0.944–0.977], P < 0.001; high intensity, 0.935 [0.911–0.959], *P* < 0.001). Finally, longer than < 30-min single session LTPA duration was associated with lower depression scores (31–60 min, 0.973 [0.952–0.995], *P* = 0.016; > 60 min, 0.974 [0.951–0.998], *P* = 0.032).

**Table 3:** Associations between Beck Depression Inventory score and variables of leisure-time physical activity

	B	95% Confidence interval	*P*
LTPA, METh/week	− 0.095	− 0.305 – 0.116	0.378
Activity level, inactive as reference			
Moderately active	0.966	0.950 – 0.983	< 0.001
Active	0.975	0.953 – 0.998	0.037
Frequency of LTPA, < 1 session/week as reference			
1–2 sessions/week	0.981	0.960 – 1.002	0.070
> 2 sessions/week	0.966	0.947 – 0.984	< 0.001
Intensity of LTPA, low intensity as reference			
Moderate intensity	0.960	0.944 – 0.977	< 0.001
High intensity	0.935	0.911 – 0.959	< 0.001
Duration of LTPA session, < 30 min as reference			
31–60 min	0.973	0.952 – 0.995	0.016
> 60 min	0.974	0.951 – 0.998	0.032

Association between depression score and leisure-time physical activity (in METh) was investigated with generalized linear regression, in all other analyses multinomial logistic regression analysis was used. All models are adjusted for age, sex, body mass index, estimated glomerular filtration rate, and current smoking. METh, metabolic equivalent of task hours, LTPA, leisure-time physical activity

We then investigated the multivariable associations between symptoms of depression and LTPA. In these analyses, similarly to the continuous BDI score, depressive symptomatology was not associated with the total amount of LTPA (Table 4). However, compared to those inactive, individuals self-reportedly moderately active (0.427 [0.284–0.644], *P* < 0.001) and active (0.546 [0.315–0.946], P = 0.031) had reduced odds of symptoms of depression. Relative to individuals reporting < 1 weekly LTPA sessions, those reporting > 2 session exhibited lower odds of symptoms depression (0.412 [0.265–0.639], *P* < 0.001). Individuals with low-intensity LTPA as reference, those reporting moderate- (0.396 [0.268–0.589], *P* < 0.001) and high-intensity LTPA (0.264 [0.144–0.485], *P* < 0.001) had reduced odds of depressive symptomatology. No differences in the odds of depressive symptoms were evident among the categories of single LTPA session duration.

**Table 4:** Associations between symptoms of depression and variables of leisure-time physical activity

	B	95% Confidence interval	*P*
LTPA, METh/week	− 1.529	− 6.573 – 3.515	0.552
Activity level, inactive as reference			
Moderately active	0.427	0.284 – 0.644	< 0.001
Active	0.546	0.315 – 0.946	0.031
Frequency of LTPA, < 1 session/week as reference			
1–2 sessions/week	0.664	0.408 – 1.083	0.101
> 2 sessions/week	0.412	0.265 – 0.639	< 0.001
Intensity of LTPA, low intensity as reference			
Moderate intensity	0.396	0.268 – 0.586	< 0.001
High intensity	0.264	0.144 – 0.485	< 0.001
Duration of LTPA session, < 30 min as reference			
31–60 min	0.642	0.382 – 1.078	0.094
> 60 min	0.585	0.331 – 1.034	0.065

Association between symptoms of depression and leisure-time physical activity (in METh) was investigated with generalized linear regression; in all other analyses, multinomial logistic regression analysis was used. All models are adjusted for age, sex, body mass index, estimated glomerular filtration rate, and current smoking. Symptoms of depression, Beck Depression Inventory score ≥ 16; METh, metabolic equivalent of task hours, LTPA, leisure-time physical activity

### Sensitivity analyses

Data on antidepressant agent purchases were available from 1206 participants (43% men, 42 [33–52] years), of whom 110 (9.1%) had such purchases. After adjustments for age, sex, BMI, eGFR, and smoking, antidepressant agent purchases were not associated with METh (Supplementary Table 1). As compared to those inactive, individuals reporting moderate activity had lower odds of having antidepressant purchases (0.608 [0.375–0.989], *P* = 0.045). The odds were also lower in those with moderate (0.511 [0.328–0.796], *P* = 0.003) or high intensity (0.360 [0.180–0.719], P = 0.004), as compared to those with low-intensity LTPA. Finally, compared to those with a single LTPA session duration < 30 min, we observed lower odds of antidepressant agent purchases in those with 31–60 min (0.517 [0.297–0.900], *P* = 0.020) or > 60 min (0.440 [0.234–0.825], *P* = 0.010) single session durations.

## Discussion

Signs of depression were associated with many aspects of LTPA in the current study of adult individuals with type 1 diabetes. Indeed, in our analyses, higher depression scores were reflected in more inactive lifestyle, lower frequency of weekly LTPA sessions, lower intensity of the physical activity, and shorter duration of the exercise sessions. Additionally, symptoms of depression measured as a BDI score ≥ 16 were associated with lower activity level, and lower frequency and intensity of the LTPA.

Our observations linking depressive mood and lower frequency of LTPA, in individuals with type 1 diabetes, are in line with those by McCarthy et al. [12] and Trief et al. [13]. However, to the best of our knowledge, beyond frequency, no prior studies have investigated other aspects of physical activity in depressive symptomatology, in adults with type 1 diabetes. In a small sample of children with type 1 diabetes, however, symptoms of depression were unrelated to physical activity, measured using the Physical Activity Questionnaire for Older Children [20]. While regular physical activity is obviously of great importance and has shown to reduce the incidence of severe diabetic retinopathy [7], other aspects of physical activity may also play important roles. Indeed, in our previous studies, intensity of the physical activity, in particular, has shown significant associations in reduced risks of diabetic long-term complications [5, 6] and premature mortality [9].

In populations other than type 1 diabetes, physical inactivity has shown to predict depression [11]. In their review of published studies on the association between physical activity and depression in type 2 diabetes, Lysy et al. concluded that individuals weekly engaging in less than 30–90 min of exercise were 72–75% more likely depressed than those more active, and those depressed were 22–90% more likely to be physically inactive than those non-depressed [21]. Nyström et al. conducted a meta-analysis of 12 studies and reported that in 10 of the included studies an improvement in depressive symptoms, following an exercise intervention was observed [22]. In the same study, the mode of activity did not impact the effect of the outcome. Based on the reviewed literature, the authors concluded that individually customized physical activity with a minimum of 30-min session time, executed with a frequency of at least thrice a week, is recommended for treating major depressive disorder [22].

A number of mechanisms linking physical inactivity and depression have been suggested. There is, for example, evidence for exercise-induced elevations in the brain concentrations of serotonin [23, 24], a neurotransmitter with a role in mood regulation [25, 26]. Increases in β-endorphin [27] and vascular endothelial growth factor [28], both linked to neurogenesis [29, 30], have also been reported to take place, following exercise. Moreover, physical activity has shown to result in a blunted cortisol response to a post-exercise stressor, suggesting a negative feedback mechanism between exercise and the hypothalamus–pituitary–adrenal axis [31]. Chronic low-grade inflammation has been shown to contribute to the pathophysiological changes associated with depressive disorders [32]. Physical activity, on the other hand, has the potential to reduce inflammation as, following exercise, a marked increase in the circulating levels of anti-inflammatory interleukin-6 has been reported [33]. In addition to the physiological mechanisms, the common symptoms of depression, including lack of motivation, fatigue, and helplessness, may further explain why depressive mood is associated with reduced physical activity. It has also been noted that individuals with diabetes and depression have fewer skills for relapse prevention and may thus need more support for continuing with exercise habits when facing various challenges in life [34]. Importantly, physical activity has shown to reduce psychological distress [35], and improve self-efficacy [36, 37] and self-esteem [37], factors that have the potential to impact mood.

Consistent with previous observations, compared to men, women more frequently exhibited symptoms of depression. While investigating the reasons behind the sex differences is beyond the scope of this paper, an interested reader will find many articles dedicated to this subject [e.g. 38–40]. In her recent contribution, Kuehner, for example, enumerated various biological, psychological, and environmental factors that could be responsible for this difference [38]. Genetics, gene-environmental interactions, hormones, and differences in physiological responses to stress were among the potential biological determinants listed. Of the psychological factors, that could be responsible for the sex difference, Kuehner mentioned neuroticism and negative affect, absence of positive affect, interpersonal orientation, rumination, and dissatisfaction related to one’s body. Finally, of the environmental factors, various early adversities, such as childhood sexual abuse which is more prevalent in girls, interpersonal violence after childhood, exposure and susceptibility to stress, and societal structural sex inequities were noted. In the future, the potential sex differences in the association between depressive symptoms and physical activity should be investigated in a sufficiently powered sample.

A large study sample and well-defined participants are important strengths of the current study. However, the present results must be considered in the context of the study design. Importantly, self-report questionnaires were used to collect data on physical activity and symptoms of depression. While both the LTPA questionnaire [41, 42] and the BDI [18] are validated methods, the potential for misreporting, and thus misclassification, remains. There may be a tendency for the participants to report in a socially acceptable way, that is, to report higher physical activity and lower symptoms of depression. To overcome the limitation of self-reporting the symptoms of depression, we additionally used register-based data on antidepressant agent purchases. Importantly, the observations made using this definition further strengthened those made when symptoms of depression were defined using BDI. However, also the use of antidepressant agent purchases is a proxy of depressive symptomatology, as not all cases are treated with medication and, beyond depression, antidepressants may be used for other indications. While we did not have data on the actual diagnoses of depression, the number of depression-related hospital visits in this sample was too small (*n* = 49) for satisfactory analyses. Another limitation has to do with patient selection, as individuals more interested in their health more likely take part in health-related studies. However, if the above is the case, it may be speculated that the current observations have likely diluted the actual phenomenon. Having included only participants with preserved renal function and no other major vascular complications, the current observations may most likely be generalized to other fairly healthy adult individuals with type 1 diabetes. Finally, the study is cross-sectional and cannot reveal causalities, but rather associations.

In conclusion, symptoms of depression are harmfully associated with LTPA in type 1 diabetes. Both depressive symptomatology and the depression score were associated with less active lifestyle, and lower frequency and intensity of LTPA. Whether increasing physical activity could improve mental well-being in type 1 diabetes should be investigated.

## Supplementary Information

Below is the link to the electronic supplementary material.

Supplementary file1 (PDF 535 kb)

## Data Availability

According to the General Data Protection Regulation of the European Union (679/ 2016), the principles of data protection should apply to any information concerning an identified or identifiable natural person and that personal data which have undergone pseudonymization, which could be attributed to a natural person by the use of additional information should be considered to be information on an identifiable natural person. Thus, according to the GDPR, all pseudonymized data are considered personal data and cannot be published openly.
